# The association between areas of secondary hyperalgesia and volumes of the caudate nuclei and other pain relevant brain structures—A 3-tesla MRI study of healthy men

**DOI:** 10.1371/journal.pone.0201642

**Published:** 2018-08-21

**Authors:** Morten S. Hansen, Mohammad S. Asghar, Jørn Wetterslev, Christian B. Pipper, Johan Mårtensson, Lino Becerra, Anders Christensen, Janus D. Nybing, Inger Havsteen, Mikael Boesen, Jørgen B. Dahl

**Affiliations:** 1 Department of Anaesthesiology, Centre of head and orthopaedics, Rigshospitalet, Copenhagen, Denmark; 2 Department of Radiology, Copenhagen University Hospital Bispebjerg and Frederiksberg, Copenhagen, Denmark; 3 Department of Anaesthesiology, Centre of head and orthopaedics, Rigshospitalet, Copenhagen, Denmark; 4 Copenhagen Trial Unit, Centre for Clinical Intervention Research, department, Copenhagen, Denmark; 5 Section of Biostatistics, Faculty of health, Copenhagen University, Copenhagen, Denmark; 6 Department of Clinical Sciences, Faculty of Medicine, Lund university, Lund, Sweden; 7 Invicro, A Konica Minolta Company, Boston, United States of America; 8 Department of Radiology and the Parker Institute, Copenhagen University Hospital Bispebjerg and Frederiksberg, Copenhagen, Denmark; 9 Department of Anaesthesiology, Copenhagen University Hospital Bispebjerg and Frederiksberg, Copenhagen, Denmark; McLean Hospital, UNITED STATES

## Abstract

**Introduction:**

Central sensitization plays a pivotal role in maintenance of pain and is believed to be intricately involved in several chronic pain conditions. One clinical manifestation of central sensitization is secondary hyperalgesia. The degree of secondary hyperalgesia presumably reflects individual levels of central sensitization. The objective of this study was to investigate the association between areas of secondary hyperalgesia and volumes of the caudate nuclei and other brain structures involved in pain processing.

**Materials and methods:**

We recruited 121 healthy male participants; 118 were included in the final analysis. All participants underwent whole brain magnetic resonance imaging (MRI). Prior to MRI, all participants underwent pain testing. Secondary hyperalgesia was induced by brief thermal sensitization. Additionally, we recorded heat pain detection thresholds (HPDT), pain during one minute thermal stimulation (p-TS) and results of the Pain Catastrophizing Scale (PCS) and Hospital Anxiety and Depression score (HADS).

**Results:**

We found no significant associations between the size of the area of secondary hyperalgesia and the volume of the caudate nuclei or of the following structures: primary somatosensory cortex, anterior and mid cingulate cortex, putamen, nucleus accumbens, globus pallidus, insula and the cerebellum. Likewise, we found no significant associations between the volume of the caudate nuclei and HPDTs, p-TS, PCS and HADS.

**Conclusions:**

Our findings indicate that the size of the secondary hyperalgesia area is not associated with the volume of brain structures relevant for pain processing, suggesting that the propensity to develop central sensitization, assessed as secondary hyperalgesia, is not correlated to brain structure volume.

## Introduction

Nociceptive stimuli can elicit sensitization of neurons in the central pain pathways.

This phenomenon of central sensitization is a manifestation of the plasticity in the central nervous system (CNS) and represents the CNS’s ability to alter and produce augmented pain responses by amplification of synaptic inputs and recruitment of subthreshold neurons. It is believed to be a contributing factor for individual pain sensitivity and may play a pivotal role in the maintenance and chronification of pain [1, 2].

Central sensitization can readily be investigated in humans with pain models utilizing either heat [3, 4], cold [5], chemical [6] or electrical [7] stimulation. Noxious heat stimulation to the skin produces primary hyperalgesia at the site of injury and secondary hyperalgesia with reduced thresholds for mechanical stimulation in the non-injured skin surrounding the injury [8–10]. Current evidence indicates that secondary hyperalgesia following a standardized heat injury is a result of central sensitization [1, 2, 11] and is expressed differently among individuals, where some individuals develop small while others develop large secondary hyperalgesia areas [9, 12]. In addition, individuals will continue to develop secondary hyperalgesia of similar magnitude when exposed to the same noxious stimuli [9, 12]. Secondary hyperalgesia is thus a robust phenomenon that may be used to phenotypically characterize individuals [9, 12], and may be used as a model to evaluate the individual level of central sensitization.

The occurrence of central sensitization in different chronic pain conditions suggests that certain individuals may be predisposed towards developing central sensitization [1, 2, 9]. An important question is why some individuals have a higher propensity for developing central sensitization, and if such individuals have a subsequent higher risk of developing pain hypersensitivity and chronic pain [1]. Currently, no sufficient explanation of the high inter-individual variance in secondary hyperalgesia areas has been provided. Understanding these variations may lead to crucial insights into central mechanisms of pain and possibly to identification of biomarkers for central sensitization.

A recent exploratory brain MRI study found structural and functional differences when comparing healthy volunteers with a small vs. large area of secondary hyperalgesia [13], demonstrating an inverse correlation of the volume of the caudate nuclei and the area of secondary hyperalgesia. The caudate nuclei are essential for the integration and control of motor, sensory, and motivational information [14]; however, studies suggest that they are also activated during pain expectancy [15], are involved in the modulation and suppression of pain [16], and are important sites for the sensory processing and spatial localization of noxious stimuli [17]. Moreover, clinical studies have indicated that reduced grey matter volume of the caudate nuclei is seen in patients with various chronic pain conditions [18–20]. Several other brain structures, including the primary somatosensory cortex, anterior and mid cingulate cortex, basal ganglia, insula and the cerebellum have been demonstrated to be intricately involved in pain processing [21, 22], illustrating the comprehensiveness of pain perception. Finally, animal and human studies have suggested that specific brainstem structures, including the rostral ventromedial medulla, the nucleus cuneiformis, and the periaqueductal gray may be involved in the development and maintenance of central sensitization [23].

The aim of the current study was to determine whether differences in brain anatomy were associated with the propensity to develop central sensitization, assessed as areas of secondary hyperalgesia. Specifically, we investigated if the size of the secondary hyperalgesia area was associated with the volume of the caudate nuclei and other brain structures relevant for pain processing.

## Materials and methods

The study was approved by the Danish Committee on Health Research Ethics for the Capital Region (H-15010473) and the Danish Data Protection Agency (RH-2015-149). In addition, the study was registered on Clinicaltrials.gov (NCT02567318).

A detailed description of the study design and methods has been published previously [24].

### Design

Briefly, the study consisted of two separate parts: Part 1: Pain testing and Part 2: MRI scans.

#### Pain testing

The isolated results from the pain testing have been presented in a separate publication [25]. In addition, these data have been used for the analyses of the present MRI data.

**Fig 1:**
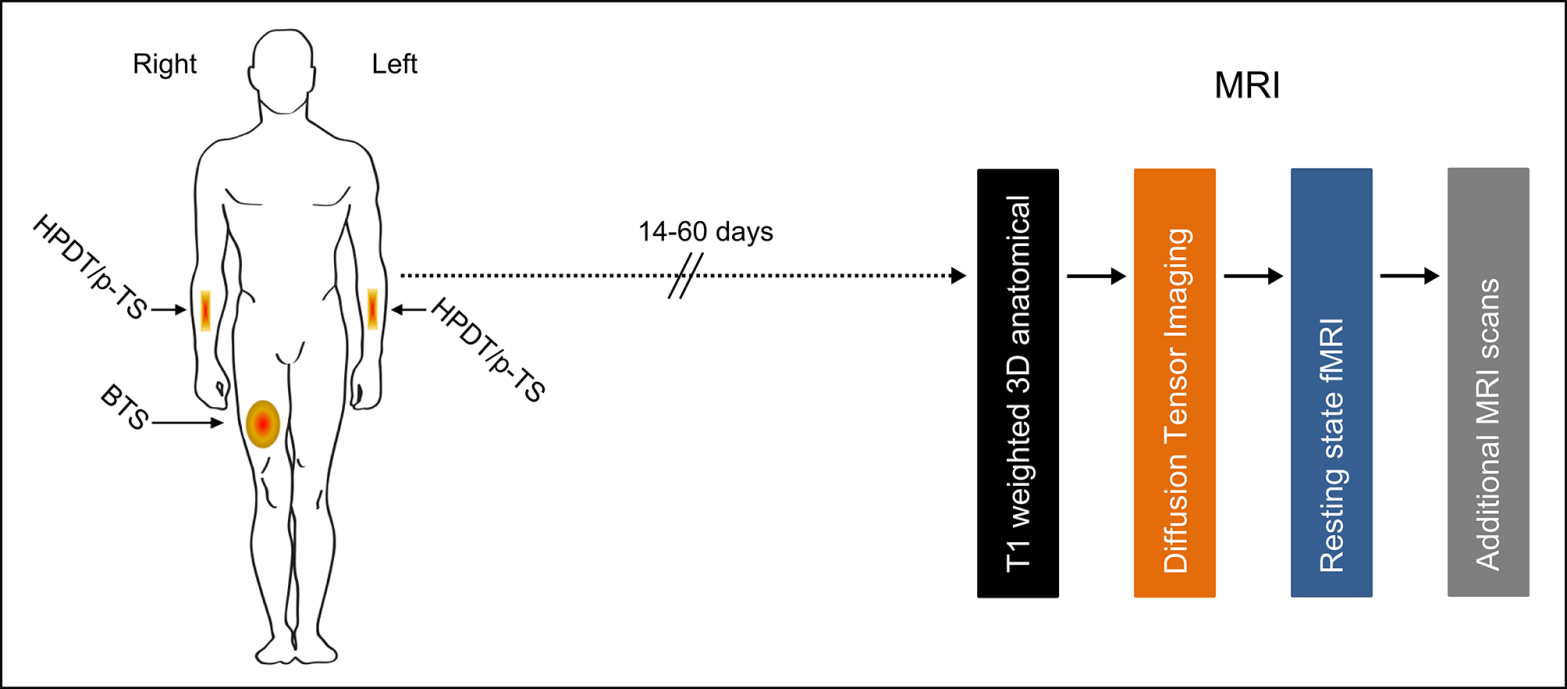
Anatomical presentation of the location of the pain testing and MRI scan sequence. Brief thermal sensitization (BTS) was conducted centrally on the anterior part of the right thigh in the midline between the anterior superior iliac spine and the base of patella. Heat pain detection threshold (HPDT) was evaluated on the anterior part of the dominant lower arm; pain during thermal stimulation (p-TS) was evaluated on the anterior part of the non-dominant lower arm. The MRI-scans were performed a minimum period of 14 days and maximum 60 days after the pain testing. The MRI scans were conducted in fixed order, starting with T1-weighted 3D anatomical scan (duration: 4 minutes) followed by diffusion tensor imaging (duration: 12 minutes), resting state fMRI scan (duration: 8 minutes) and additional scans of technical or diagnostic character. Total duration of MRI scan sequence was approximately 50 minutes. Abbreviations: MRI, Magnetic resonance imaging; fMRI, functional Magnetic resonance imaging.

The pain testing was conducted at a minimum of 14 days and at a maximum of 60 days prior to the MRI scans to avoid any carry-over effects. For details regarding the pain testing please see the published protocol [24]. Briefly, all included participants were tested with the following pain models (Fig 1):

**Brief thermal sensitization (BTS)**:

A computer-controlled thermode (Somedic MSA Thermotester; size 2.5 x 5 cm.) was placed on the upper right thigh. The thermode was then heated to 45°C for three minutes. Afterwards the assessment of secondary hyperalgesia (see below) was conducted while the 45°C heated thermode remained on the skin of the participant [4, 9, 12, 26]. The assessment took approximately 1–2 minutes, resulting in a maximum duration of the heat stimulation of 5 min.

**Assessment of secondary hyperalgesia**:

The area of secondary hyperalgesia was quantified after stimulation with a monofilament (Von Frey hair) with a nominal value of 18 (bending force 490 mN) in 4 linear paths arranged in 90° around the center of the thermode. Stimulation began well outside the area of secondary hyperalgesia, and advanced in 5 mm/sec intervals towards the center of the thermode. When clear change in sensation occurred (intense burning, pricking, tenderness) the location was marked, and the longitudinal and transverse axes were measured for rectangular area calculation [4, 8, 9, 12].

**Heat pain detection threshold**:

The individual heat pain detection threshold (HPDT) was evaluated by placing the thermode on the anterior part of the dominant lower arm. The temperature of the thermode was then increased by 1°C/second from a baseline of 32°C, until the participant perceived the heat as painful and pressed a button. The HPDT was estimated as an average of 4 separate stimulations with an interval of 6–10 seconds [8, 27].

**Pain during thermal stimulation (p-TS)**:

The thermode was placed on the lower non-dominant arm, and was heated to 45°C for 1 min. During the 1 min. heating the participant evaluated the pain using the electronic Visual Analog Scale (VAS) (Somedic USB-VAS), with an index of 0–100 mm, where 0 mm represented “no pain”, and 100 mm represented “worst pain imaginable”. A VAS area under the curve (VAS-AUC) and a maximum VAS-score was calculated by the computer software [8, 27].

#### MRI scans

On the day of the MRI scans each included participant underwent multimodal whole brain MRI scans (Fig 1); no other tests or assessments were conducted on this study day. The total duration of the MRI scans was approximately 50 min. Following completion of MRI-scans, all images were reviewed by an experienced radiology consultant. In the case of suspected pathological findings, the participant was informed hereof and was referred to a specialist in neurology for further examination.

**MRI data acquisition and imaging protocols**:

All MRI scans were performed with a Siemens MAGNETOM Verio 3-tesla MRI scanner, with b17 software, and a 32-channel head coil.

**Anatomical images**:

Anatomical images were obtained using a T1-weighted 3D FLASH (160 sagittal slices, matrix 256x256 mm, Field of view 256 mm, echo time (TE) 2.98 ms, repetition time (TR) 2300 ms, Slice 1 mm, in plane resolution 1x1 mm, flip angle 9°).

**Additional MRI sequences not analysed**:

We also performed the following MRI sequences: Diffusion tensor imaging, resting state epi single shot functional MRI, arterial spin labelling, b0 field maps, T2-FLAIR, T2-weigthed TSE sequence, and GRE hemo sequences. Due to technical problems we were not able to use the DTI-scans for analysis in this study, and consequently the secondary and exploratory outcome measures described in the published study protocol could therefore not be evaluated at present. As reported in the published study protocol [24], results from the resting state functional MRI will be reported in a separate paper.

The remaining MRI scans were either of technical character or for diagnostics and will per protocol not be reported in this paper.

#### Physiological measurements

Pulse frequency, respiration frequency, and end-tidal PCO_2_ were measured during the entire scan session, including before and after the resting state scan.

#### Psychological testing

The participants were tested with two separate psychological tests. The participants completed the psychological tests prior to the pain testing and the MRI scans.

*Pain Catastrophizing Scale* (PCS) is a 13-point questionnaire on a five-point Likert scale with values from 0–4. The highest achievable score is 52, and the PCS can be subdivided in 3 sections that evaluate Rumination, Magnification, and Helplessness [28].

*Hospital Anxiety and Depression Scale* (HADS) is a 14-point questionnaire on a four-point Likert scale with values ranging from 0–3. The highest achievable score is 53, and the HADS can be subdivided in two sections that evaluate Anxiety and Depression [29].

### Setting

All MRI scans were conducted at the Department of Radiology, Bispebjerg and Frederiksberg Hospitals, Copenhagen, Denmark. The pain testing [25] was conducted at the department of Anaesthesiology, Rigshospitalet, Copenhagen, Denmark. Data was collected in the period from October 2015 to December 2015. All analyses were conducted at the department of Anaesthesiology, Rigshospitalet, and at the Section of Biostatistics, Faculty of health, Copenhagen University, Denmark.

### Study participants

Healthy male volunteers age 18–35 years, who had participated in preceding pain testing [25] were included in the study. Oral and written informed consent was obtained from all participants prior to inclusion in the study. The participants received EUR 67 (USD 74) for their participation in the study. Inclusion and exclusion criteria are listed in Table 1.

**Table 1:** Inclusion and exclusion criteria.

Inclusion criteria	Exclusion criteria
Age ≥18 years and ≤35 years Speak and understand the Danish language Male sex Signed informed consent Participation and completion of the study: “Heat pain detection threshold is associated with the area of secondary hyperalgesia following brief thermal sensitization: a study of healthy male volunteers” [19]	Inability to cooperate to the test Weekly intake of >21 units of alcohol, or intake of >3 units of alcohol within 24 hours before study day Substance abuse, assessed by the investigator Consummation of analgesics within 3 days before study day Consummation of antihistamines within 48 hours before study day Consummation of antidepressant medication within 30 days before the study day Consummation of prescription medicine within 30 days before the study day Consummation of coffee or caffeine within 24 hours before study day. Neurological illnesses Chronic pain Psychiatric diagnoses Eczema, wounds or sunburns on the sites of stimulation Body Mass Index of >30 kg/m^2^ or <18 kg/m^2^. Unwilling to receive information regarding potential pathological findings in relation to the MRI. Any kind of trauma resulting in pain and administration of analgesics in the period between pain testing and MRI scan. Head trauma in the period between the pain testing and the MRI. Contraindications to MRI (claustrophobia, pacemaker implant, artificial heart valve, cochlear/stapes prosthetics, irremovable insulin pump, neuro-stimulator, metal from previous surgery, metallic foreign objects, catheters, shunts, draining tubes, and surgical procedures within the last 6 weeks (subjected to individual evaluation)).

Abbreviations: MRI, Magnetic resonance imaging.

### Outcome measures

#### Primary analysis

To investigate the association between the volume of the left and right caudate nuclei and the magnitude of the area of secondary hyperalgesia induced by brief thermal sensitization.

#### Secondary analyses

To investigate the association between the magnitude of the area of secondary hyperalgesia and cortical as well as subcortical brain structures relevant for pain processing (primary somatosensory cortex, anterior and mid cingulate cortex, putamen, nucleus accumbens, globus pallidus, insula and the cerebellum).

#### Exploratory analyses

To investigate the association between the volume of the left and right caudate nuclei and the following five parameters: 1. HPDT; 2. p-TS max. VAS-score; 3. p-TS VAS-AUC; 4. PCS; and 5. HADS scores.

To investigate possible neuroanatomical differences between participants displaying a small area of secondary hyperalgesia (lower quartile) compared to participants displaying a large area of secondary hyperalgesia (upper quartile). The same cortical and subcortical brain structures as specified in the primary and secondary analyses were included in the analysis.

### Sample size analysis

Sample size estimation was based on a Z-test of the Fisher transformed Pearson correlation with results from a previous study [13]. With a true correlation of R = - 0.4 between the area of secondary hyperalgesia and the volume of the caudate nuclei, and with a significance level of 2.5–5% according to the single step method, a sample size of 52 was needed to obtain a power of 0.80 (β = 0.20). Our sample size estimation was based on results from a study were only participants that produced small or large area of secondary hyperalgesia were included. In the present study we aimed to include participants without prior knowledge of their areas of secondary hyperalgesia and thus also expected inclusion of participants with intermediary size areas of secondary hyperalgesia. To secure a reasonable high sample size when comparing the upper and lower quartiles based on area of secondary hyperalgesia we aimed to include 120 participants.

### Structural MRI preprocessing

Anatomical T1W-weighted images were preprocessed and analyzed using the FreeSurfer imaging analysis suite version 5.3, which is freely available for download online (http://surfer.nmr.mgh.harvard.edu/) and is a semi-automatic software that performs volumetric segmentation of cortical and subcortical structures [30–32]. Cortical volumes were extracted according to the Desikan-Killiany cortical atlas [33]. To avoid possible confounding due to inter-participant head size differences, all volumes were adjusted for intracranial volume using a method based on the analysis of covariance approach outlined by Raz et al. [34]. All volumes were extracted to a spread sheet for separate data analysis.

### Statistical analyses

Individual levels of secondary hyperalgesia, HPDT, p-TS VAS-max, and p-TS VAS-AUC was obtained as Estimated Best Linear Unbiased Predictors (EBLUPS) [25].

#### Primary analysis

To adequately estimate inter-individual differences in secondary hyperalgesia areas the area of secondary hyperalgesia was adjusted for body surface area. Individual body surface areas were calculated using the Mosteller formula [35]. The association between the volume of the left and right caudate nucleus and the magnitude of the secondary hyperalgesia area was estimated by multiple linear regression. The ability to predict the size of the secondary hyperalgesia area by measurement of the caudate nuclei volume was quantified by R^2^. P-values were adjusted for multiple testing using the single step method [36].

#### Secondary analyses

The association between the magnitude of the secondary hyperalgesia area and the volume of the cortical and subcortical brain structures relevant for pain processing (primary somatosensory cortex, anterior and mid cingulate cortex, putamen, accumbens nucleus, globus pallidus, insula and the cerebellum) was estimated by multiple linear regression. Model reduction was performed by backwards elimination with a 5% cut-off level.

#### Exploratory analyses

The association between the volume of the left and right caudate nucleus and HPDT, p-TS VAS-max, p-TS VAS-AUC, PCS and HADS respectively was evaluated per exploratory outcome by multiple linear regression. The findings were adjusted for multiple testing using the single step method [36].

Possible neuroanatomical differences between participants displaying a small area of secondary hyperalgesia (lower quartile) and participants displaying a large area of secondary hyperalgesia (upper quartile) were estimated using unpaired t-test. The findings were adjusted for multiple testing using the single step method [36].

#### Sensitivity analyses

Four sensitivity analyses were performed to assess the robustness of the findings.

In the first sensitivity analysis further adjustments by age, weight, BMI, and MAP were performed. To adjust for hand-dominance a second sensitivity analysis were performed where only right-handed participants were included. To adjust for difference in ethnicity a third sensitivity analysis where performed where only ethnic Scandinavians were included. To evaluate the impact of individual body surface, we conducted a fourth sensitivity analysis where we did not adjust for body surface area.

#### Post-hoc analyses

A post-hoc analysis was conducted to investigate the association between the area of secondary hyperalgesia and brain structures not included in the per-protocol analyses, but with a possible relevance for pain processing. Thus, the association between the magnitude of the secondary hyperalgesia area and the volume of the left and right amygdala, hippocampus, and thalamus were estimated by multiple linear regression. Moreover, when comparing participants with a small and large area of secondary hyperalgesia possible differences in the volume of these three structures were estimated using unpaired t-test. P-values were adjusted for multiple testing using the single step method; however, P-values in the per-protocol planned analyses were not adjusted further by inclusion of the additional brain structures in the post-hoc analysis.

P-values corresponded to Wald-tests and *P<0*.*05* were evaluated as significant.

All statistical analyses that were not computed by the MRI software were calculated using the open-source statistical programming environment R (R Core Team (2014). (R: A language and environment for statistical computing. R Foundation for Statistical Computing, Vienna, Austria. URL http://www.R-project.org/).

## Results

**Fig 2:**
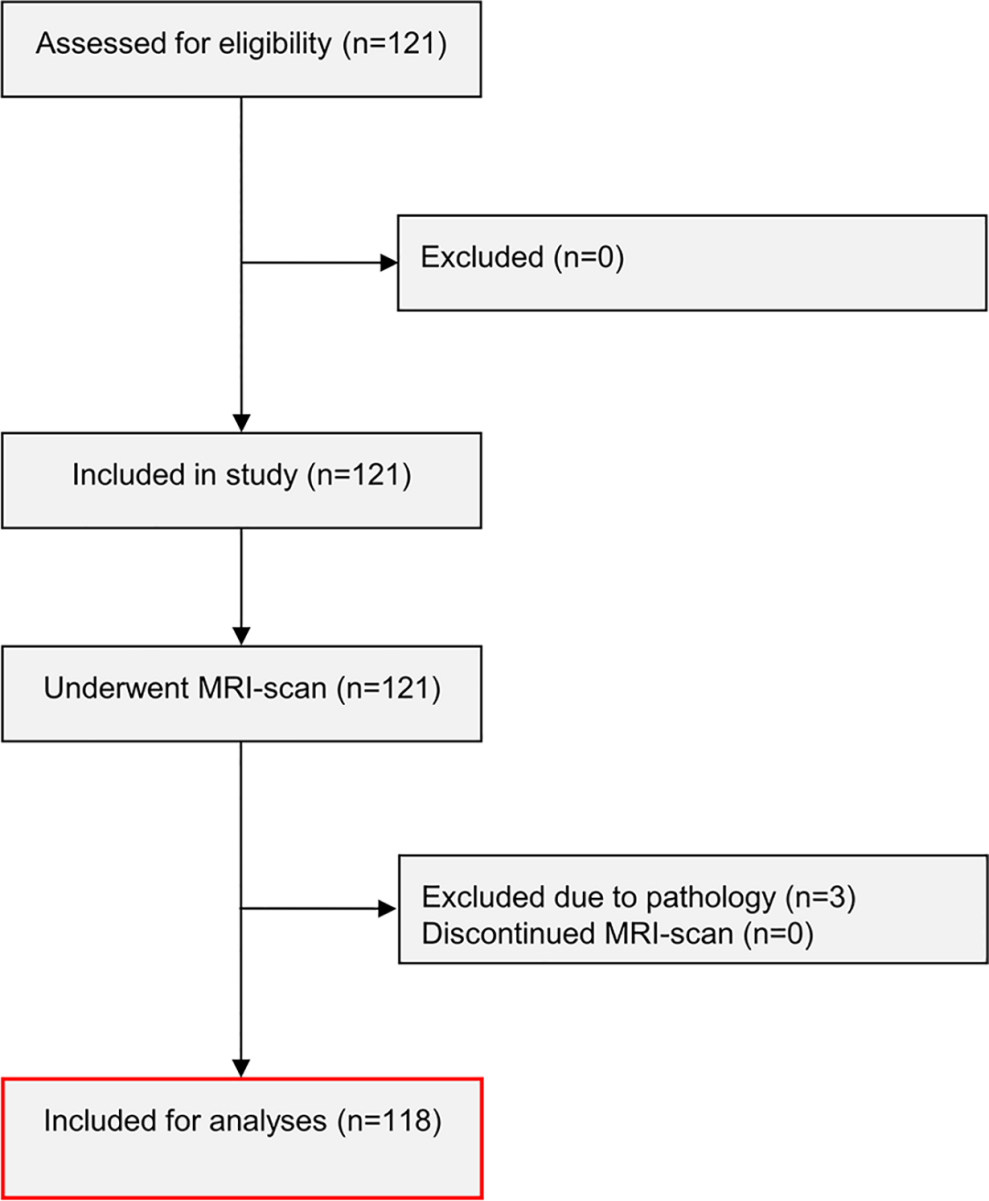
Flowchart of included study participants. 121 participants were assessed for eligibility and included in the study. 3 participants were excluded due to pathological findings following magnetic resonance imaging (MRI), and consequently 118 participants were included in the final analysis.

121 healthy participants were included in the study. All participants completed the MRI-scans, but following clinical review, 3 participants were excluded due to suspected pathological findings. Thus, 118 participants were included in the final analysis (Fig 2). Of the 118 included participants, 10 were left-handed, and 15 had one or more parents with non-Scandinavian ethnicity. The median interval between the completion of the preceding study session with pain testing and the MRI scan was 17 days (interquartile range (IQR) 16–18).

The median size of the secondary hyperalgesia area was 448 cm^2^ (IQR 346–528) with a range of 135–789 cm^2^ (Table 2).

**Table 2:** Basic characteristics, pain testing results and psychological test results of the 118 participants included in the analysis.

Variable	Median (IQR)	Range (min-max)
Age (years)	22 (20–24)	18–33
Height (m)	1.84 (1.79–1.88)	1.68–2.03
Weight (kg)	76.8 (70.0–84.8)	57–110
BMI (m^2^/kg)	22.82 (21.02–24.51)	18.12–28.63
MAP (mm Hg)	90 (84–96)	73–117
Heart rate (bpm)	64 (58–70)	46–97
Pain testing results		
Area of secondary hyperalgesia (cm^2^)	448 (346–526)	135–789
HPDT (°C)	45.57 (43.78–46.60)	38.70–51.01
p-TS VAS-max (mm)	33.5 (18.79–53.41)	2.41–95.99
p-TS VAS-AUC	1151 (648–1850)	83–4456
Psychological test results		
PCS-helplessness	4 (2–6.25)	0–17
PCS-rumination	5 (3–8)	0–12
PCS-magnification	3 (1–4)	0–10
PCS-total	12 (7–17)	1–31
HADS-anxiety	4 (2–6)	0–13
HADS-depression	1 (1–3)	0–16
HADS-total	6 (3–8.25)	0–21

All medians and ranges of the area of secondary hyperalgesia, heat pain detection thresholds and pain during thermal stimulation have been estimated by calculating the estimated best linear unbiased predictors (EBLUPS). Test results of PCS and HADS were extracted following completion of all MRI-scans.

Abbreviations: IQR, Interquartile range; BMI, body mass index; BPM, Beats per minute; MAP, mean arterial pressure; HPDT, heat pain detection threshold; p-TS, pain during thermal stimulation; VAS-max, maximum visual analogue scale; VAS-AUC, visual analogue scale area under the curve; PCS, Pain Catastrophizing Scale; HADS, Hospital Anxiety and Depression Scale; IQR.

Basic characteristics for the 118 participants and evoked pain results extracted from the preceding study session are displayed in Table 2. No adverse or serious adverse events were reported.

### Secondary hyperalgesia and caudate nuclei

**Fig 3:**
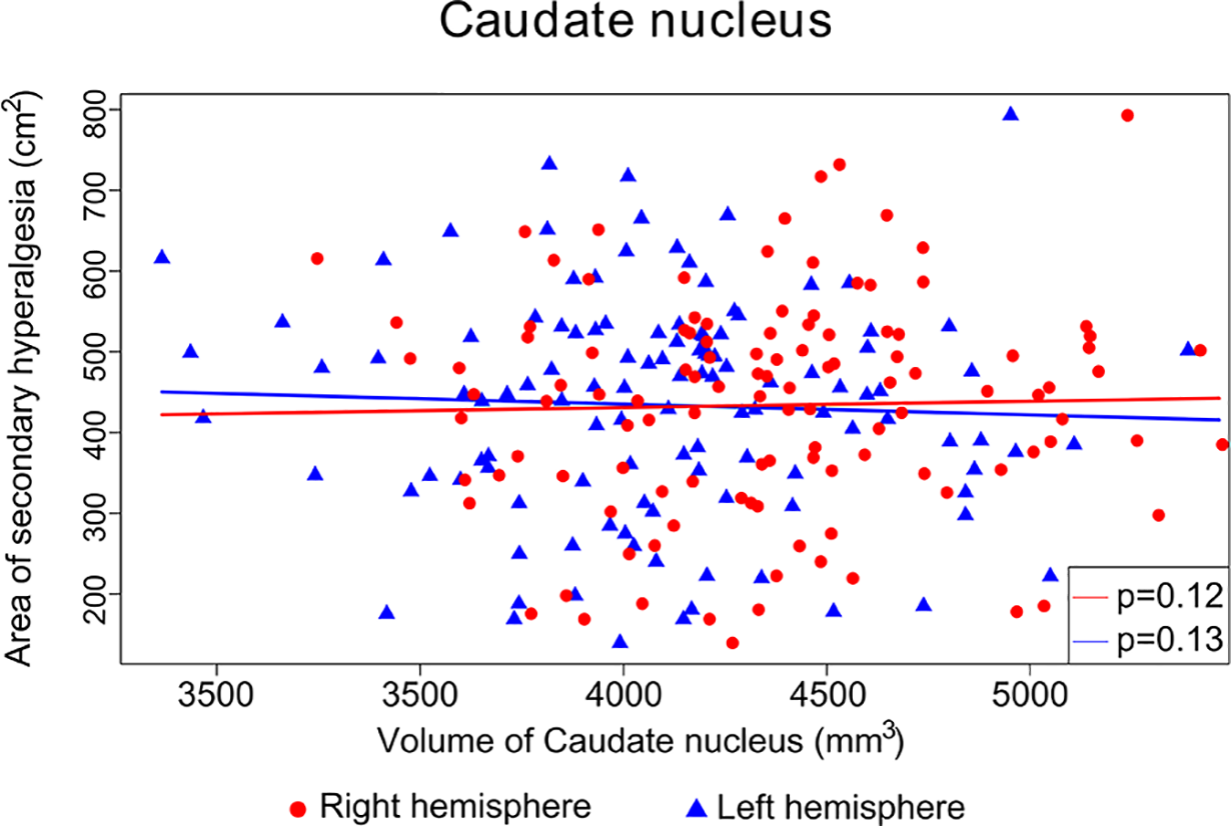
Associations between the area of secondary hyperalgesia and the volume of the caudate nuclei. Scatter plot of the volume of the right (red dots) and left (blue triangles) caudate nucleus and the corresponding area of secondary hyperalgesia of each included participant. Individual volumes of the caudate nuclei were adjusted for intracranial volume, and individual areas of secondary hyperalgesia were adjusted for body surface area. Regression lines demonstrate no significant association between area of secondary hyperalgesia and the volume of the right and left caudate nucleus (right hemisphere, p = 0.12, left hemisphere, p = 0.13).

We found no significant associations between the volume of the right and left caudate nucleus and the size of the area of secondary hyperalgesia (right hemisphere, single-step adjusted *p* = 0.13, left hemisphere, single-step adjusted *p* = 0.12). The adjusted R^2^ was estimated to 0.0068, and our regression analyses demonstrated that a one-mm^3^ increase in the volume of the right caudate nucleus resulted in an estimated increase of 0.103 cm^2^ in secondary hyperalgesia area (with a family-wise adjusted 95% confidence interval (95% CI) of (-0.028 to 0.233)). Likewise, a one-mm^3^ increase in the volume of the left caudate nucleus resulted in an estimated decrease of -0.107 cm^2^ in secondary hyperalgesia area (95% CI (-0.239 to 0.025)) (Fig 3).

### Secondary hyperalgesia and cortical and subcortical areas

**Fig 4:**
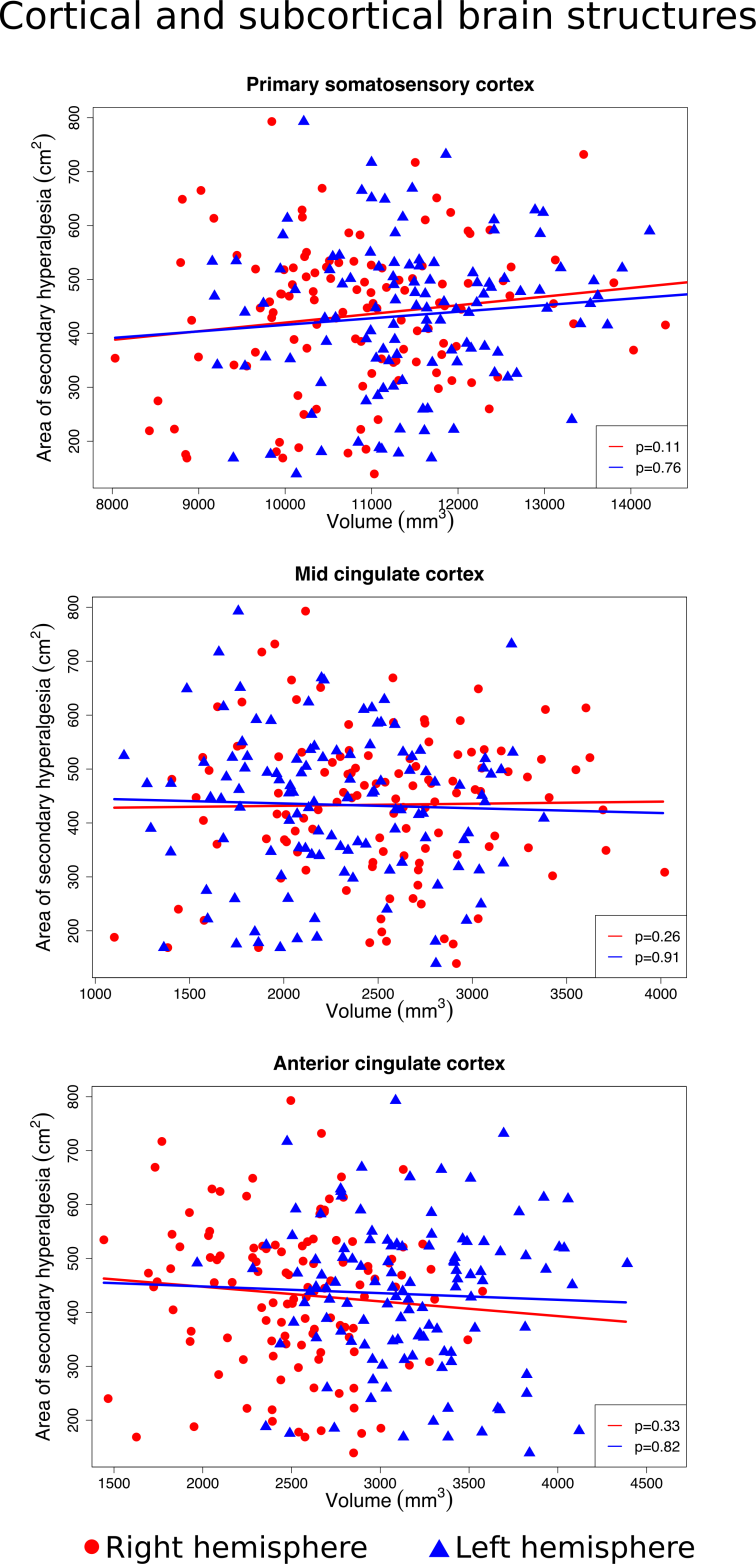
Associations between the size of the secondary hyperalgesia area and the volume of the primary somatosensory cortex, mid-, and anterior cingulate cortex. Scatter plots of individual volume measurements of the brain structures belonging to the right hemisphere (red dots) and left hemisphere (blue triangles). Volumes of individual brain structures were adjusted for intracranial volume, and individual areas of secondary hyperalgesia were adjusted for body surface area. Red and blue regression lines and p-values ≥0.05 demonstrate no significant association between the size of the secondary hyperalgesia area and the volume of the primary somatosensory cortex, the mid- and anterior cingulate cortex.

**Fig 5:**
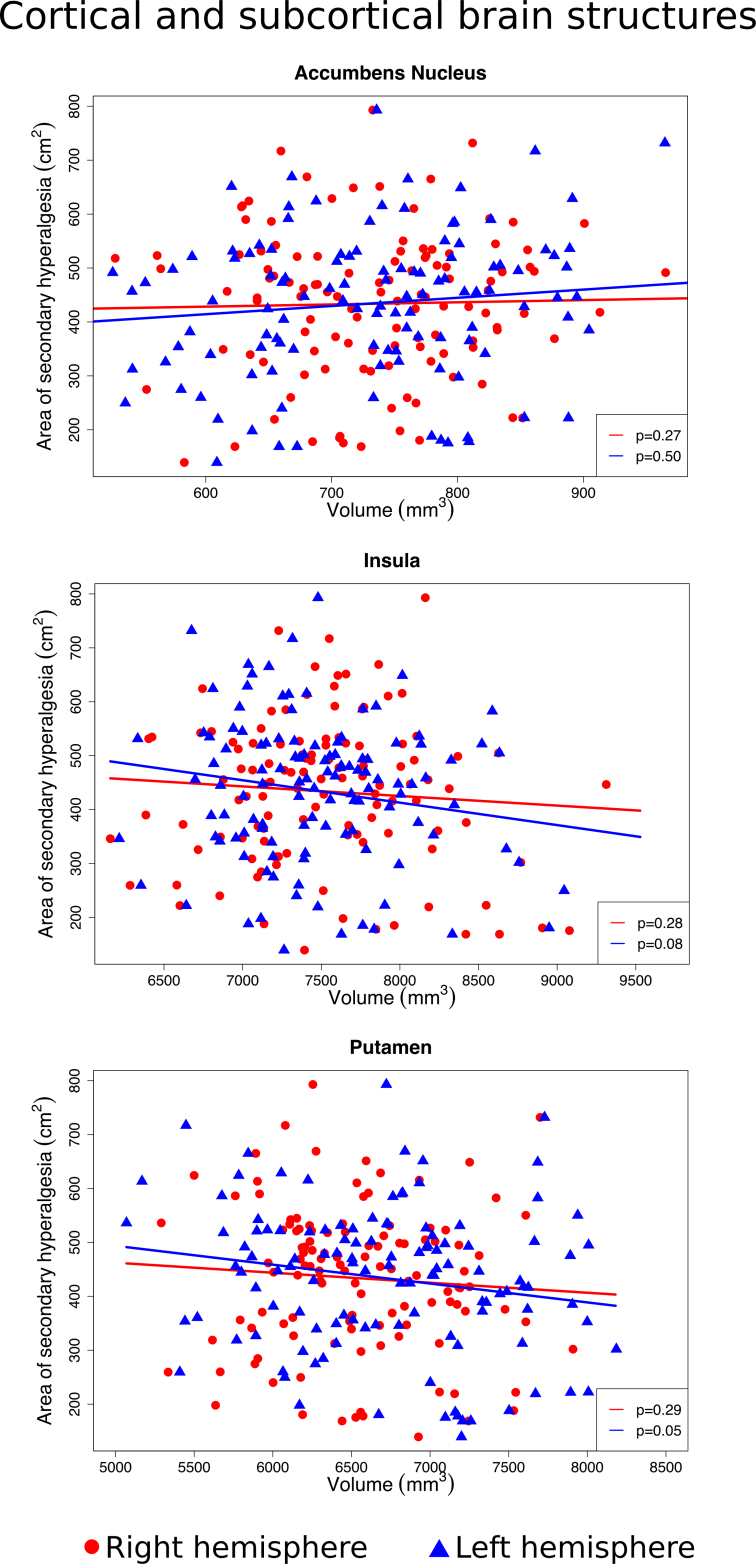
Associations between the size of the secondary hyperalgesia area and the volume of the accumbens nucleus, insula, and the putamen. Scatter plots of individual volume measurements of the brain structures belonging to the right hemisphere (red dots) and left hemisphere (blue triangles). Volumes of individual brain structures were adjusted for intracranial volume, and individual areas of secondary hyperalgesia were adjusted for body surface area. Red and blue regression lines and p-values ≥0.05 demonstrate no significant association between the size of the secondary hyperalgesia area and the volume of the accumbens nucleus, insula and the putamen.

**Fig 6:**
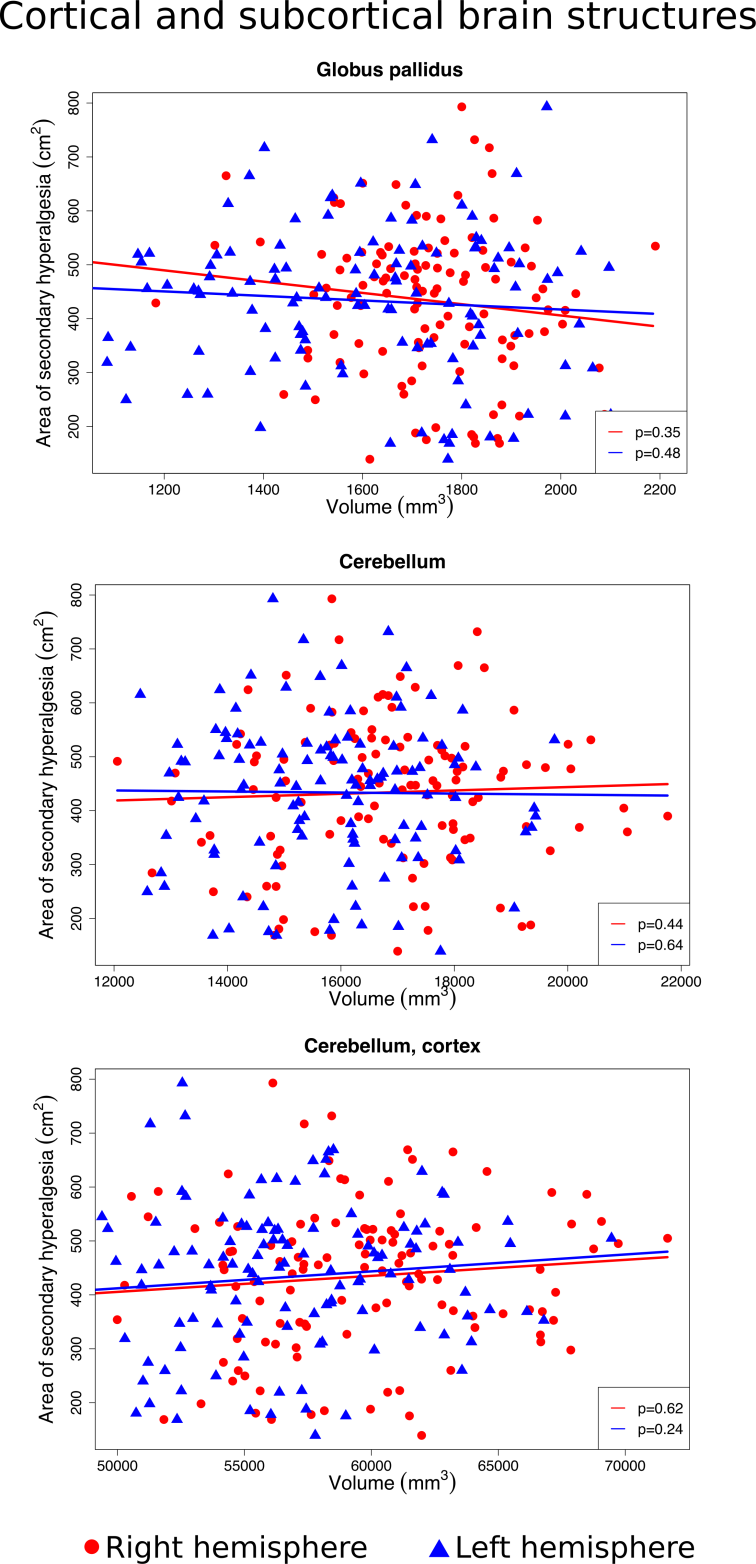
Associations between the size of the secondary hyperalgesia area and the volume of the globus pallidus, the cerebellum, and the cortex of the cerebellum. Scatter plots of individual volume measurements of the brain structures belonging to the right hemisphere (red dots) and left hemisphere (blue triangles). Volumes of individual brain structures were adjusted for intracranial volume, and individual areas of secondary hyperalgesia were adjusted for body surface area. Red and blue regression lines and p-values ≥0.05 demonstrate no significant association between the size of the secondary hyperalgesia area and the volume of the globus pallidus and the cerebellum.

We found no significant association between the size of the area of secondary hyperalgesia, and the volume of the primary somatosensory cortex (right hemisphere *p* = 0.11, left hemisphere *p* = 0.76), anterior cingulate cortex (right *p* = 0.33, left *p* = 0.82) and mid cingulate cortex (right *p* = 0.26, left *p* = 0.91), putamen (right *p* = 0.29, left *p* = 0.05), nucleus accumbens (right *p* = 0.27, left *p* = 0.5), globus pallidus (right *p* = 0.35, left p = 0.48), insula (right *p* = 0.28, left *p* = 0.08) or the cerebellum’s white matter (right *p* = 0.44, left *p* = 0.64) and cortex (right *p* = 0.62, left *p* = 0.24) (Figs 4–6).

In the post-hoc analyses we found no significant associations between the size of the secondary hyperalgesia area and the volume of the amygdala (right *p* = 0.96, left *p* = 1), hippocampus (right *p* = 0.99, left *p* = 1), and thalamus (right *p* = 0.96, left *p* = 0.96).

### Caudate nuclei and pain testing results

We found no significant associations between the volume of the right and left caudate nuclei and HPDT (right caudate nucleus *p* = 1, left caudate nucleus *p* = 1), p-TS VAS-max (right *p* = 1, left *p* = 1) or p-TS VAS-AUC (right *p* = 1, left *p* = 1).

### Caudate nuclei and Hospital Anxiety and Depression Scale (HADS) and Pain Catastrophizing Scale (PCS)

We found no significant associations between PCS and the volume of the caudate nuclei (right caudate nuclei *p* = 0.96, left caudate nuclei *p* = 0.94) or HADS and the caudate nuclei (right *p* = 0.26, left *p* = 0.24) score.

### Small vs. Large area of secondary hyperalgesia

Following stratification based on areas of secondary hyperalgesia, the median area size in the groups including the lower (N = 29) and upper quartile (N = 29) was 261 cm^2^ (IQR 203–319) and 579 cm^2^ (IQR 516–629) respectively (Table 3).

**Table 3:** Results from pain testing and psychological testing of the upper and lower quartile based on magnitude of secondary hyperalgesia area adjusted for body surface.

Characteristic	Small area (lower quartile)	Large area (upper quartile)
Number of participants (n)	29	29
Area of secondary hyperalgesia (cm^2^)	261 (203–319)	579 (516–629)
HPDT (°C)	46.32 (45.56–47.11)	43.34 (41.93–44.59)
p-TS VAS-max (mm)	28.15 (17.03–41.56)	49.44 (26.74–70.61)
p-TS VAS-AUC (mm^2^)	1002.46 (565.96–1407.50)	1719.7 (991.2–2801.0)
PCS-helplessness	3 (1.25–6.5)	4 (2.25–6.75)
PCS-rumination	5 (3–7)	5 (4–7)
PCS-magnification	2.5 (1–4)	2.5 (1–4)
PCS-total	11 (6.25–16.75)	12.5 (9–16.75)
HADS-anxiety	3 (2–6)	4.5 (3–6.75)
HADS-depression	1 (0–2)	2 (1–4)
HADS-total	4 (2.25–7.5)	7 (4.25–9)

Numbers are reported in median and interquartile ranges.

All medians and ranges of area of secondary hyperalgesia, heat pain detection thresholds and pain during thermal stimulation have been estimated by calculating the estimated best linear unbiased predictors (EBLUPS).

Abbreviations: IQR, interquartile range; HPDT, heat pain detection threshold; p-TS, pain during thermal stimulation; VAS-max, maximum visual analogue scale; VAS-AUC, visual analogue scale area under the curve; min, minimum; max, maximum; PCS, Pain Catastrophizing Scale; HADS, Hospital Anxiety and Depression Scale.

**Fig 7:**
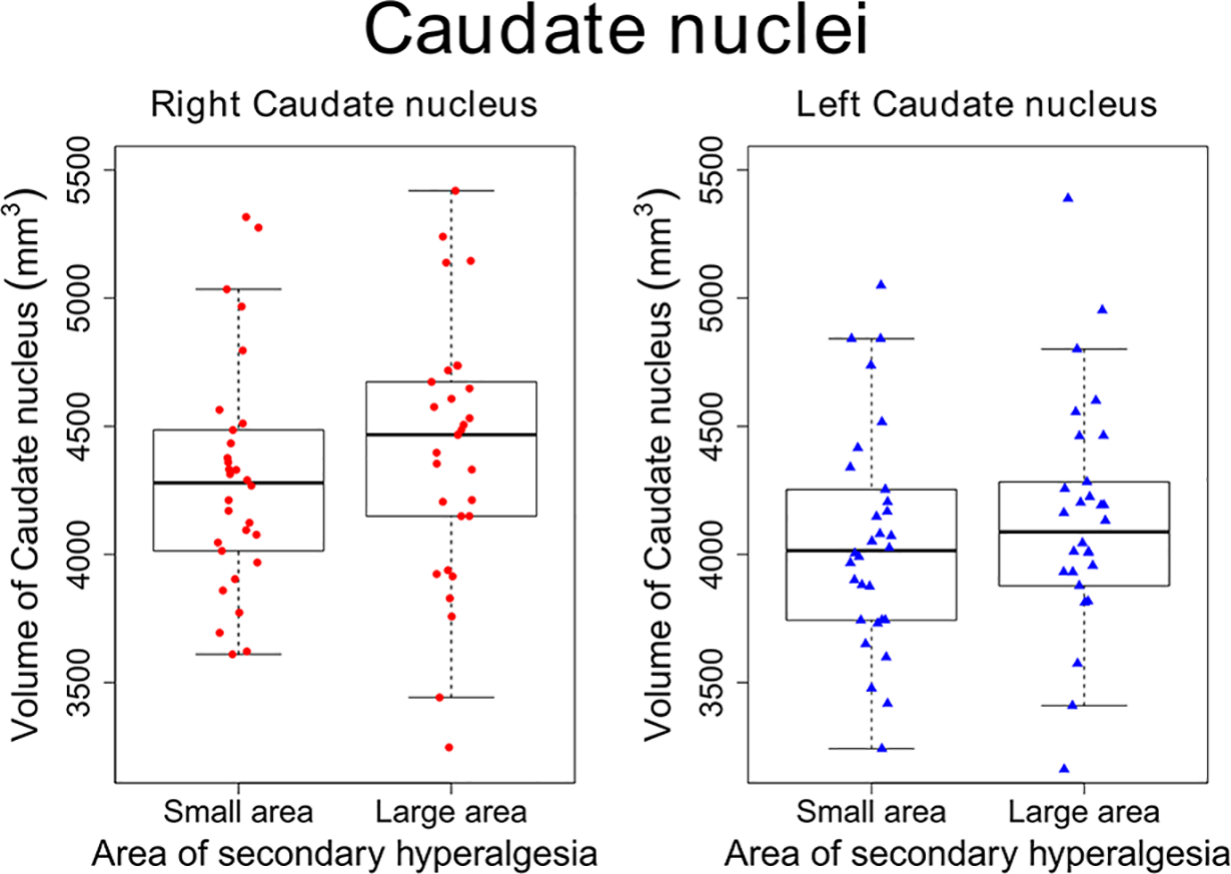
Volumes of caudate nuclei when comparing participants with a small versus large area of secondary hyperalgesia. Boxplot of the right (dots) and the left (triangles) volumes of the caudate nuclei corresponding to the participants with small (lower quartile) and large (upper quartile) areas of secondary hyperalgesia respectively. Points correspond to individual volume measurements, the thick horizontal line corresponds to median volume of the caudate nuclei, and whiskers indicate borders of 1.5 times the upper or lower quartile. There was no significant difference between the groups (small vs large areas of secondary hyperalgesia) regarding the volume of the caudate nucleus in either the left (p = 1) or the right (p = 1) hemisphere.

When comparing participants with a small area of secondary hyperalgesia (lower quartile, N = 29) vs. participants with a large area (upper quartile, N = 29) we found no significant differences in the volumes of the caudate nucleus (right *p* = 1, left *p* = 1) (Fig 7), the primary somatosensory cortex (right *p* = 0.91, left *p* = 1), anterior cingulate cortex (right *p* = 0.991, left *p* = 1) and mid cingulate cortex (right *p* = 1, left *p* = 1), putamen (right *p* = 1, left *p* = 0.75), nucleus accumbens (right *p* = 1, left *p* = 0.98), globus pallidus (right *p* = 1, left *p* = 1), insula (right *p* = 1, left *p* = 0.93),or the cerebellum’s white matter (right *p* = 1, left *p* = 1) and cortex (right *p =* 0.98, left *p* = 1).

Likewise, in the post-hoc analyses we did not detect any significant differences in volumes of the amygdala (right *p* = 1, left *p* = 0.99), hippocampus (right *p* = 0.98, left *p* = 1) and thalamus (right *p* = 0.89, left *p* = 0.99) when comparing participants with a small vs. large area of secondary hyperalgesia.

### Sensitivity analyses

Adjustment of age, weight, BMI and MAP did not demonstrate different results when comparing to our primary analysis. Secondly, we found that exclusion of left-handed participants or participants with non-Scandinavian ethnicity did not change the results markedly. Lastly, applying secondary hyperalgesia areas without adjusting for body surface area did not change the results markedly.

## Discussion

The major question addressed by this study is whether differences in the propensity to develop secondary hyperalgesia and thus central sensitization are related to differences in the volume of brain structures in healthy volunteers.

We found that phenotypic expression of secondary hyperalgesia was not associated with differences in the volume of the caudate nuclei, nor was it associated with differences in the volumes of the primary somatosensory cortex, anterior and mid cingulate cortex, putamen, nucleus accumbens, globus pallidus, insula, cerebellum, amygdala, hippocampus and thalamus.

The occurrence of pain is dependent on both peripheral mechanisms and the excitability of the central nervous system. Sensitization of the central nervous system is characterized by enhanced responsiveness of nociceptive neurons to normal or subthreshold afferent inputs and is believed to play an important role in various pain conditions such as osteoarthritis, fibromyalgia, headache and neuropathic pain [1, 2].

Even though central sensitization was characterized more than three decades ago, its pathophysiology still remains elusive. It is believed that alterations in synaptic efficacy, membrane excitability, transmission inhibition, as well as changes in microglia, astrocytes, and gene transcription leads to the changes in functional properties that are characteristic for central sensitization [1, 2]. Clinically, the effects of central sensitization can be observed as enhanced temporal summation, allodynia, hyperalgesia, and after-sensations (perception of a stimulus after the stimulus has been terminated) [2]. One essential feature of central sensitization is secondary hyperalgesia, i.e. expansion of receptive fields enabling input from non-injured tissue to be perceived as painful [1].

Previous studies have indicated a link between the magnitude of secondary hyperalgesia area and persistent pain. Patients suffering from fibromyalgia or rheumatoid arthritis display larger areas of secondary hyperalgesia compared to healthy individuals [37, 38]. Moreover, following iliac crest bone harvest [39] and after abdominal surgery [40, 41] and thoracotomy [42], a correlation was demonstrated between increasing size of secondary hyperalgesia area and the development of persistent pain. Interestingly, no correlation was found between the magnitude of secondary hyperalgesia and surgical characteristics (length of incision, volume of deep tissue trauma and nerve lesion severity), which suggests that the size of the secondary hyperalgesia area reflects individual predispositions to develop central sensitization [39]. One study found no correlation between pre-surgical areas of secondary hyperalgesia and postoperative pain following arthroscopy [43]. However, in this study secondary hyperalgesia was assessed to predict pain 1–10 days postoperatively and not to predict persistent or chronic pain.

In the present study we used high-resolution MRI at 3-Tesla to investigate if volume estimates of brain structures involved in pain processing would correlate to the area of secondary hyperalgesia induced by a thermal stimulus. MRI permits precision measurement and detection of minute differences in brain structure [44, 45]. We found no significant association between areas of secondary hyperalgesia and the volume of the caudate nuclei. This is emphasized by the estimated R^2^, indicating that only 0.0068% of the variation of in secondary hyperalgesia area is explained by the volume of the caudate nuclei. Moreover, we found no significant associations between heat pain detection thresholds or pain during thermal stimulation and the volume of the caudate nuclei, indicating that cutaneous heat pain sensitivity is also not related to the volume of the caudate nuclei. Finally, we found no significant associations between the area of secondary hyperalgesia and the volume of any other pain relevant brain structures.

Our findings indicate that the predisposition for central sensitization, assessed as secondary hyperalgesia area, is not related to brain structure volume, and that individual levels of central sensitization are not determined by cortical or subcortical structural differences.

In contrast, a previous exploratory MRI study reported a correlation between the volume of the caudate nuclei and the area of secondary hyperalgesia [13]. In both the present and the former study, the neuroanatomy of healthy participants was examined by 3-Tesla MRI. However, there are important differences between the two studies: Firstly, as opposed to the former study, we examined predefined anatomical areas of interest (brain structures related to pain processing [21, 22]) and corrected our data for total body surface in the analysis of the present data. Secondly, in the present study we included a high number of healthy male participants (N = 121), without prior knowledge of their individual areas of secondary hyperalgesia, as compared to inclusion of fewer participants of both gender (N = 40) based on the magnitude of the secondary hyperalgesia area (and with a disproportionally higher number of females in one group) in the study by Asghar et al. [13]. The difference in method of inclusion is especially important since it may have contributed significantly to the differences in results. Inclusion based on area of secondary hyperalgesia increases the inter-participant differences in development of secondary hyperalgesia, and may produce more visible results; however we believe that our approach in the current study is more robust since it is strictly driven by the hypothesis, and not data driven. Nonetheless, this may have resulted in a lower inclusion of participants with small or large areas of secondary hyperalgesia and increased the risk of type 2 errors.

We performed the MRI scans on average 17 days after the pain testing to avoid carry-over effects. It has been demonstrated that areas of secondary hyperalgesia following BTS remain stable over a period of minimum 4 weeks [12]. This allowed for investigation without the risk of recording neuroanatomical changes due to recent or repetitive pain stimulations. We included a large number of participants (n = 121) making this the largest MRI study of secondary hyperalgesia reported so far. There were no missing data and no protocol violations. Moreover, we based our primary and secondary outcome measures on known cortical and subcortical brain structures relevant for pain and central sensitization [13, 21, 22]. We conducted separate sensitivity analyses to test the robustness of our findings and did not find different results compared to our primary analysis. Finally, based on the high number of included participants combined with a stringent methodological approach we believe our findings to be robust and of high quality.

Studies of healthy participants have indicated that reduced grey matter volume of pain relevant structures is correlated with increased visceral sensitivity [46] as well as increased heat pain sensitivity [47]. Results from the present study are not coherent with those findings. However, heat pain detection thresholds have been demonstrated only to offer moderate explanation of the inter-individual variations in secondary hyperalgesia [25]; suggesting that cutaneous heat pain sensitivity and areas of secondary hyperalgesia represent two distinctively different pain entities. Moreover, to the authors’ knowledge, no studies have investigated the association between visceral sensitivity and secondary hyperalgesia areas.

Several studies of chronic pain patients have identified neuroanatomical correlates of chronic pain [18, 20, 48] and reported reduced grey matter volumes in multiple pain relevant brain structures. However, comparisons between structural abnormalities found in pain free healthy individuals, and chronic pain patients have limited value, since evidence suggest that structural grey matter abnormalities observed in chronic pain patients are a result of experience-dependent neuronal plasticity, and that these abnormalities are reversible when the pain stimulus is terminated [48–51]. In support of this, a study of healthy individuals reported that the initial MRI-scans did not show structural differences between individuals characterized with high and low pain sensitivity, but following repetitive noxious stimulation the high pain sensitizers were more prone to develop grey matter density reductions [44]. This suggests that healthy individuals with high innate pain sensitivity are more prone to develop structural abnormalities comparable to chronic pain patients, but also that pain sensitivity is not influenced by the structural anatomy of the brain.

The present study has some limitations. Firstly, we applied strict inclusion criteria resulting in a homogenous population of healthy male participants. Inclusion of both sexes would have introduced several variations such as possible neuroanatomical differences related to sex [52, 53], hormonal influence of the menstrual cycle on MRI findings [54, 55], and possible interaction between the menstrual cycle and pain responses [56]. Moreover, the BTS method has only been validated in healthy male volunteers [12]. Secondly, due to limitations in the software and the ancillary anatomical atlas we were not able to segment the secondary somatosensory cortex, the supplementary motor area, the substantia nigra and the subthalamic nucleus as we had specified in our published study protocol [24]. Finally, we included participants regardless of hand-dominance and ethnicity. MRI-studies often include right-handed participants only, additionally, ethnicity may influence pain thresholds [57]. Our sensitivity analyses, however, did not show any differences in findings when excluding left-handed or non-Scandinavian participants illustrating the robustness of our results.

In conclusion, we did not find significant associations between the area of secondary hyperalgesia induced by a thermal injury, and the volume of the caudate nuclei or any other predefined brain structures involved in pain processing, indicating that the propensity to develop central sensitization is not correlated to the volume of pain related brain structures.

We suggest that future studies of contributing factors to central sensitization should include investigations of the functional connectivity of the CNS, the endogenous opioid system, relevant molecular mechanisms, and psychological factors. Potential findings in future studies may shed light on the etiology of central sensitization and ultimately provide us with novel pharmaceutical targets in the treatment of acute and chronic pain.

## Supporting information

S1 Table CONSORT 2010 checklist. (DOC)
